# Application of Magnetic Resonance Imaging in the Evaluation of Nutritional Status: A Literature Review with Focus on Dialysis Patients

**DOI:** 10.3390/nu13062037

**Published:** 2021-06-14

**Authors:** Tsutomu Inoue, Eito Kozawa, Masahiro Ishikawa, Hirokazu Okada

**Affiliations:** 1 Department of Nephrology, Faculty of Medicine, Saitama Medical University, Saitama 350-0495, Japan; t_inoue@saitama-med.ac.jp; 2 Department of Radiology, Faculty of Medicine, Saitama Medical University, Saitama 350-0495, Japan; 8kozawa@saitama-med.ac.jp; 3 School of Biomedical Engineering, Faculty of Health and Medical Care, Saitama Medical University, Saitama 350-1241, Japan; ishikawa@saitama-med.ac.jp

**Keywords:** magnetic resonance imaging, diffusion tensor imaging, arterial spin labeling, blood oxygenation level-dependent, nutritional status, dialysis patients

## Abstract

Magnetic resonance imaging (MRI) is indispensable in clinical medicine for the morphological and tomographic evaluation of many parenchymal organs. With varied imaging methods, diverse biological information, such as the perfusion volume and measurements of metabolic products, can be obtained. In addition to conventional MRI for morphological assessment, diffusion-weighted MRI/diffusion tensor imaging is used to evaluate white matter structures in the brain; arterial spin labeling is used for cerebral blood flow evaluation; magnetic resonance elastography for fatty liver and cirrhosis evaluation; magnetic resonance spectroscopy for evaluation of metabolites in specific regions of the brain; and blood oxygenation level-dependent imaging for neurological exploration of eating behavior, obesity, and food perception. This range of applications will continue to expand in the future. Nutritional science is a multidisciplinary and all-inclusive field of research; therefore, there are many different applications of MRI. We present a literature review of MRI techniques that can be used to evaluate the nutritional status, particularly in patients on dialysis. We used MEDLINE as the information source, conducted a keyword search in PubMed, and found that, as a nutritional evaluation method, MRI has been used frequently to comprehensively and quantitatively evaluate muscle mass for the determination of body composition.

## 1. Introduction

### 1.1. The Origins of Medical Magnetic Resonance Imaging (MRI)

Medical magnetic resonance imaging (MRI) was established in the 1970s as a tomographic imaging method for the human body, and its full-scale clinical implementation began in the 1980s. Since then, the technology of medical imaging hardware has progressed by leaps and bounds; the development of versatile imaging methods has likewise been actively pursued. Currently, MRI has become an indispensable diagnostic imaging method in the medical field.

### 1.2. Principles and History of MRI

MRI is based on the physical phenomenon of nuclear magnetic resonance (NMR). In some types of nuclei, such as the hydrogen nucleus (referred to as “proton” or “1H” as it is a nucleus that consists solely of a proton; the number “1” indicates the mass number and the atomic number is often omitted), which rotates along the axis of rotation, the + charge of the nucleus generates a magnetic field. When a strong magnetic field in a certain direction is applied to the nucleus, it starts to rotate with an angular momentum called “Larmor precession”, that is, a cone about the direction of the magnetic field, and each nucleus has its own frequency. When radio waves of the same frequency as the precession are applied to the nucleus, a resonance phenomenon called NMR causes radio waves to absorb energy. Once the radio waves are turned off after the resonance phenomenon, the nucleus returns to its original state while releasing energy. By capturing this energy released as an electrical signal, an NMR signal is obtained. NMR is a technique used to investigate molecular structures, various intermolecular interactions, and molecular motion states and is used in a wide range of fields, such as polymer chemistry, biochemistry, and medicine.

In comparison to NMR, which has zero-dimensional information with no positional identification, MRI can be defined as a positionally-identified NMR measurement method. There is a law that states that the resonant frequency of an atom is proportional to the strength of the magnetic field. By combining a strong magnetic field with magnetic fields of different strengths, depending on the position (a gradient magnetic field), it was discovered that any position in space could be accurately determined using the strength of the magnetic field, and this NMR phenomenon became the principle of tomography. This discovery occurred 30 years after that of NMR. Although it uses the resonance phenomenon of atomic nuclei, it has nothing to do with radioactivity; thus, the term MRI came to be used idiomatically without the N in NMR.

The MRI technology has progressed dramatically since its introduction into clinical practice, and the number of clinical applications has increased accordingly, driving the development of the technology further. The principles and imaging methods of medical MRI have been documented previously [1]. The advantages of MRI include the absence of ionizing radiation, good soft-tissue contrast with multiple imaging methods, and the ability to reconstruct any cross-section with similar image quality, based on the imaging principle. This is limited to not only two-dimensional but also three-dimensional reconstruction. In addition, because the basic principle, the magnetic resonance (MR) signal, depends on various physical parameters, MRI can display morphological and structural information, as well as provide a wide range of functional information using diffusion, perfusion, and frequency decomposition techniques. Currently, clinical MRI observes water molecules (mainly consisting of hydrogen atoms), which is the most abundant constituent of living organisms, although it is also possible to collect signals from carbon and sodium atoms. Due to this diversity of imaging methods, the clinical applications of MRI are not just limited to use in neurology or psychiatry, but rather extend to the examination of the abdominal organs, heart, musculoskeletal system, and body fat.

### 1.3. MRI and Nutrition Research

The role of MRI in nutrition includes the measurement of organ volumes, analysis of constituent components (hard tissues, such as bones; soft tissues, such as fat and muscles), and acquisition of location information, such as the distribution of these components, through its function as a tomographic imaging method. Because of the objective, quantitative, and predominantly non-invasive nature of MRI, repeated evaluations are possible, and changes after interventions can be assessed. In the past, nutritional assessments using MRI were focused mainly on the fat and muscle mass; thus, there was no significant difference in the subject of assessment between an MRI and an X-ray computed tomography (CT) scan, except for the fact that no ionizing radiation was used in MRI.

However, with the introduction of high magnetic fields in clinical MR machines and advances in analytical technology, imaging methods exclusive to MRI technology are now being used in the field of nutrition, such as functional MRI (fMRI), e.g., blood oxygenation level-dependent (BOLD) MRI, diffusion-weighted imaging (DWI) methods, including diffusion tensor imaging (DTI), and MR spectroscopy (MRS). In view of this technical and historical background about the advances in MRI technology, this article presents a literature review of the clinical applications of fMRI in nutrition research.

## 2. Materials and Methods

A literature search was conducted using PubMed for the medical database MEDLINE, produced by the National Library of Medicine (NLM). The search was conducted on 28 December 2020. Articles published before the year 2000 were not included in the study.

First, we searched for studies in which MRI was used to assess the nutritional status of patients on dialysis. To prevent missing out on relevant articles, we used a free word search using the following search terms in all the fields: “magnetic resonance imaging,” “nutrition,” and “dialysis”. Thirty-two published papers were identified from the search. Of these, only nine reported the use of MRI for nutritional assessment (the others reported its use for the diagnosis of comorbidities or complications) and were suitable for our review; thus, there were limitations of the evidence, and we opined that if we were to conduct a systematic review, the scarce number of studies limited only to patients on dialysis would severely underestimate the relevance of MRI as a tool for the evaluation of nutritional status. Contrastingly, there were many research papers on the use of MRI in the assessment of nutritional indices if we did not limit the search only to patients on dialysis, and we opted for a literature review study design. 

Therefore, we conducted a search for articles, in which MRI was the main topic of the study, as “magnetic resonance imaging [MeSH Major Topic]”. We then searched for articles that included “nutrition” as a free word in the abstract or title to narrow down the target articles. Furthermore, we searched only for research papers targeting humans (“nutrition” [Title/Abstract] AND “magnetic resonance imaging” [MeSH Major Topic] AND “humans” [Filter]). Case reports were excluded from the study. Consequently, 68 articles were found, and their titles and abstracts were reviewed. We excluded papers where MRI was used to detect diseases, that is, where MRI was performed for a purpose different from nutritional evaluation. However, research reports with MRIs of the brain or organs were incorporated if they were intended for nutritional evaluation. For the review, we also searched for relevant articles in the reference lists of the included articles. Only articles in English were included in the study.

## 3. Results and Discussion

### 3.1. Summary of Search Results

MRI is mainly used in clinical medicine for tomographic imaging of living organisms; however, in nutritional research, its most common use was found to be the analysis of body composition, such as muscle and adipose tissue. In a nutritional assessment, the excellent soft-tissue contrast of an MRI was used to evaluate the body composition of the human body. In addition to soft-tissue imaging, MRI was performed to evaluate the volume of abdominal organs and the brain. This method takes advantage of the MRI as an imaging test; however, a MRI, in addition to tomography, is capable of extracting a variety of biological information by devising an imaging sequence. BOLD MRI, diffusion-weighted MRI, DTI, arterial spin labeling (ASL), magnetic resonance elastography (MRE), and MRS were considered to be either non-morphological MRI or fMRI based on the content of the articles and reviews included in this literature review (Table 1). The following are some observations drawn from specific examples from the search results.

### 3.2. MRI for Structural Evaluation: MRI-Based Assessment of Body Fat Distribution

**Figure 1:**
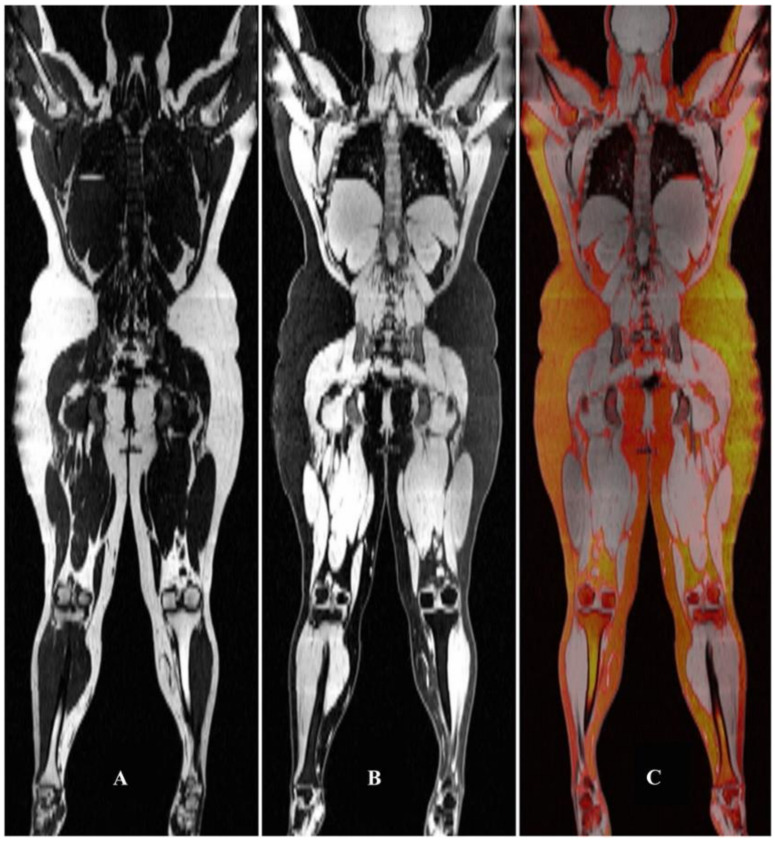
Separated fat (**A**) and water (**B**) MRI of an obese individual. Panel (**C**) depicts the fat overlayed in color on the water grayscale image. From Seabolt et al. (2015), with permission.

Assessment of body fat composition could be performed using imaging techniques, including radiography, CT, and MRI (Figure 1) [44,45]. MRI was reported to accurately and reliably assess body fat distribution and characteristics [46,47]. T1-weighted imaging was reported to differentiate between the proton signals from water and fat owing to their different T1 relaxation times. Adipose tissue appeared bright on T1-weighted images, and this feature was used for the quantification of subcutaneous adipose tissue, visceral adipose tissue, bone marrow fat, and intermuscular adipose tissue. In addition, T1-weighted imaging was used to assess muscular fat infiltration in neuromuscular disorders [2,48]. The use of MRI for the evaluation of adipose tissue was reviewed and reported [49].

In patients on dialysis, albumin and prealbumin were reported to be associated with nutritional and inflammatory statuses. Molfino et al. [3] evaluated the contribution of adiposity to prealbumin levels in patients receiving dialysis. Of 48 patients receiving hemodialysis, the total skeletal muscle mass and visceral and subcutaneous adipose tissues were measured using MRI. Prealbumin was positively associated with visceral adipose tissue and negatively associated with interleukin-6 (IL-6). In contrast, albumin was positively associated with the normalized protein catabolic rate (nPCR) and negatively associated with IL-6, but not with any measure of adiposity. Prealbumin, similar to albumin, was associated with markers of nutrition (nPCR) and inflammation, although, unlike albumin, prealbumin levels were positively associated with visceral adiposity [3].

**Figure 2:**
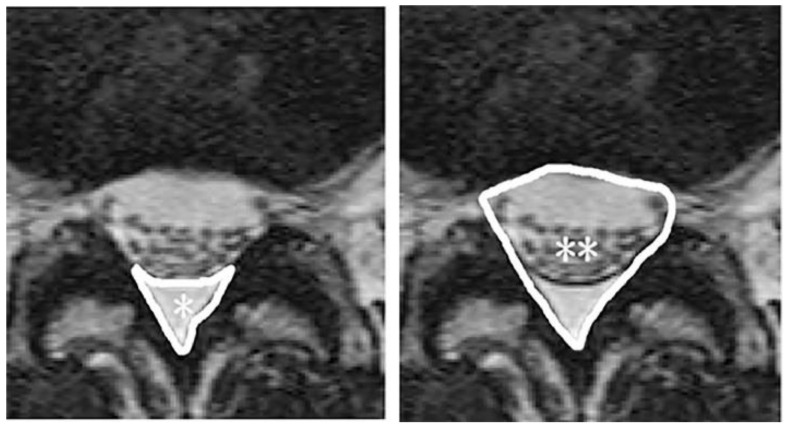
Cross-sectional area of epidural fat (EF) * and the spinal canal **. From Ishihara et al. (2019), with permission.

Regarding other reports not limited to patients on dialysis, two studies were reviewed that investigated the relationship between diet and the volume of adipose tissue in various locations (for example: visceral, subcutaneous abdominal, and trunk adipose tissues), and MRI was used to quantify the adipose tissue [4,5]. There has also been a report of fatty deposits in the liver that were assessed by MRI on the basis of the liver signal intensity [50]. These reports aimed to compare the associations of dietary patterns with liver fat contents. Moreover, there is an interesting report about fat tissue in the epidural space (epidural fat (EF), Figure 2) [6]. Overt accumulation of EF, referred to as spinal epidural lipomatosis (SEL), can compress the spinal cord, leading to the development of neurological symptoms, such as lower back pain. MRI is useful for the evaluation of EF; studies in which MRI was used have shown that it is statistically significantly associated with metabolic syndrome (Table 2) [6] and visceral and liver fat deposition [6,7,8].

### 3.3. MRI for Structural Evaluation: Evaluation of Muscle Mass by MRI

Because the resonance frequency of protons in water and in fat is different, MRI can distinguish between the two; therefore, MRIs can provide high contrast between fat and water, which allows for an accurate assessment of muscle mass. Skeletal muscle mass and function were reported to be negatively affected by a variety of conditions inherent to chronic kidney disease (CKD) and dialysis treatment in a study. Skeletal muscle mass and function served as indicators of the nutritional and clinical state of patients with CKD, and low values or derangements over time were strong predictors of poor patient outcomes [9]. However, muscle size and function can be affected by different factors, may decline at different rates, and may have different patient implications. Therefore, operational definitions of frailty and sarcopenia have emerged to encompass these two dimensions of muscle health, that is, size and functionality.

Johansen et al. [10] investigated 38 patients on dialysis and 19 healthy sedentary controls and used an MRI of the lower leg to determine the total cross-sectional area and the area of contractile and non-contractile tissues of the ankle dorsiflexor muscles. Patients on dialysis were less active and walked more slowly than the control subjects. The total muscle compartment cross-sectional area was not significantly different between patients on dialysis and the healthy controls; however, the contractile cross-sectional area was smaller in the dialysis patients even after adjustment for age, sex, and physical activity. Significant atrophy and increased non-contractile tissue were present in the muscles of patients undergoing dialysis [10].

Another research group used MRI to accurately assess muscle mass [11]. They recruited 105 adult participants on maintenance hemodialysis. The psoas, paraspinous, and mid-thigh muscle areas were measured using an MRI, and the lean body mass was measured using a dual-energy absorptiometry scan. The results showed that the psoas, paraspinous, and mid-thigh muscle areas were associated with an increase in lean body mass. The psoas muscle area provided a better measure of whole-body muscle mass than the paraspinous muscle area but was a slightly inferior measurement to the mid-thigh measurement. This study showed that, in body composition studies, a single axial MRI at the L4–L5 level can be used to provide information on both fat and muscle.

Gamboa et al. [12] used MRI to investigate whether the combination of nutritional supplementation and resistance exercise would have additive effects on muscle mass. They found that six months of nutritional supplementation during hemodialysis increased the muscle protein net balance and mid-thigh fat area. Three months of nutritional supplementation also increased the markers of mitochondrial content in the muscle. They concluded that the study was underpowered for the detection of differences; the combination of nutritional supplementation and exercise failed to show further benefit in protein accretion or muscle cross-sectional area [12]. Other studies have used MRI as a method to measure muscle mass in patients on dialysis [13,14].

Wells et al. [15] studied the relationship between total body protein and the cross-sectional skeletal muscle area in liver cirrhosis using MRI and found that overhydration influenced the skeletal muscle area. Although this report was not a study on dialysis patients, it provided interesting suggestions that need to be taken into consideration when measuring muscle mass in dialysis patients with excess fluid volume.

### 3.4. MRI for Structural Evaluation: Simultaneous Evaluation of Multiple Tissues by MRI

As mentioned above, because MRI depicts water and fat separately with good contrast, it is also possible to assess muscle mass and fat components simultaneously. Similar body compositions were obtained in a comparative evaluation using MRI and the bioimpedance method [16].

A similar study was reported in patients on dialysis [17]. This study used multi-frequency bioimpedance spectroscopy (BIS) of the arm and whole body to estimate muscle mass and subcutaneous adipose tissue in patients receiving hemodialysis by comparing these results with those of MRI. Total body and arm muscle mass and subcutaneous adipose tissue were measured using MRI. Correlations between MRI and the BIS model were high for the arm and whole body subcutaneous adipose tissues and arm and whole-body muscle mass. The results of this study indicated that total body muscle mass and subcutaneous adipose tissue can be predicted accurately, using arm BIS models with the advantages of convenience and portability, and it could be useful in assessing the nutritional status of hemodialysis patients.

MRI can also quantify the fatty components in the bone marrow [51], liver, and muscle. A study used quantitative MRI to assess yearly disease progression in patients with facioscapulohumeral muscular dystrophy type 1 [52]. The MRI Dixon technique (a T1-weighted imaging method for fat suppression with capabilities of enhancing the contrast between water and fat) was used to evaluate muscle fat replacement. The result showed that MRI detected the progression of the disease, often before changes could be appreciated in strength and functional tests. Considering the ability of MRI to measure fat and muscle simultaneously, the authors expected that MRI will also be useful in accurately classifying sarcopenia. It is noteworthy that the results of the MRI evaluation significantly correlated with a clinical diagnosis of normal obesity, sarcopenia, and sarcopenic obesity [18].

### 3.5. MRI for Structural Evaluation: Measurement of the Size of Organs by MRI

There have been reports of MRI being used to measure the volume of various organs accurately, including the brain. Conventional brain MRI can also be a useful method to study the relationship between the nutritional status and the degree of brain degeneration and atrophy.

A study, not conducted on patients with CKD, reported that malnutrition and lower vitamin B1 and B12 levels were independently associated with a risk of white matter hyperintensities in brain MRI [19]. There are studies that have performed simultaneous assessment of the brain and skeletal muscle or brain and fat. Bourdel-Marchasson et al. [20] evaluated both the brain and muscles of participants using a T1-weighted MRI. Sarcopenia features were more frequent in frail subjects than in prefrail subjects and were associated with a decrease in gray matter volumes involved in motor control [20].

### 3.6. Diffusion-Weighted MRI and Diffusion Tensor Imaging

DWI is an MRI technique that utilizes the diffusion phenomenon caused by the Brownian motion of water molecules in tissues. It was first reported by Le Bihan in 1986 [53]. It is now an indispensable imaging method for the diagnosis of acute cerebral infarction. Cellular edema is induced in acute cerebral infarction, and the extracellular fluid space becomes narrow. Therefore, the movement of water molecules in the extracellular fluid space is restricted, and the signal is higher than that of the surrounding brain parenchyma. Similarly, in cancerous tissues, the cell density is high, and the intercellular space is narrowed, resulting in a strong high-intensity signal. The apparent diffusion coefficient can be calculated from the results of imaging with two or more different b-values; although this coefficient is a quantitative value of DWI, it is relative to the image and not standardized.

While the apparent diffusion coefficient map of DWI is useful for assessing acute strokes and malignancies, its use in the field of nutritional science is less common. DTI, an extension of DWI technology, is a means of assessing nerve fiber structure in the white matter and spinal cord [54] and is often used to investigate the relationship between nutrition and the cranial nerves.

The DWI is an imaging method that documents the degree of diffusion of water molecules due to thermal motion. Diffusion usually occurs in a disordered manner, that is, if there is no structure that hinders diffusion, the diffusion direction of a certain proton is three-dimensionally equivalent in all directions and is spherical. This is called isotropic diffusion. However, when there is a structure that restricts the movement of protons, the direction of diffusion is biased, which is called anisotropic diffusion. Taking the central nervous system as an example, nerve fibers are regularly arranged in the same direction in the white matter. In such a structure, it can be considered that protons diffuse easily along the axons but not in the direction across the thick lipid-covered nerve fibers. The anisotropic diffusion index is called the fractional anisotropy (FA) value. Using this principle, a method called DTI was devised for imaging the course of nerve fibers in the brain and spinal cord.

In a study that compared brain structures using DTI in patients on hemodialysis to individuals without known kidney disease, using FA and mean diffusivity, patients on hemodialysis had a significantly lower FA across multiple white matter fiber tracts. Similarly, patients on hemodialysis had significantly higher mean diffusivity in multiple anterior brain regions. In patients on hemodialysis, white matter disease in the anterior parts of the brain is more common than in the posterior parts compared to that in controls without kidney disease. This pattern of injury is similar to that observed in aging, suggesting that developing CKD, and finally, kidney failure, may result in a phenotype consistent with accelerated aging [21].

A research report, not limited to only patients on dialysis, evaluated the beneficial effects of long-chain omega-3 polyunsaturated fatty acids (LC-n3-FA) on white matter microstructural integrity based on the FA value [22]. Other similar studies reported that breast milk feeding in low-birth-weight infants was associated with increased FA values in the white matter, which implied an improved structural connectivity of developing networks in the white matter [23,24]. This means that feeding of breast milk is linked to improved neurodevelopmental outcomes. In addition to breast milk, MRI studies have shown that adequate caloric intake, including fat, during infancy is important for the volume of cortical gray matter and the degree of white matter development, which was evaluated by DTI [25]. Using DTI, the influence of alcohol use during adolescence on white matter microstructure was investigated [26]. Decreased FA was found in moderate-to-heavy drinking men, which suggested the association between alcohol use and neural development.

As described above, DTI can be used to assess nutrition, brain development, and atrophy through the evaluation of brain microstructure and is considered to be a particularly useful tool for obtaining information on white matter.

### 3.7. Arterial Spin Labeling

Perfusion is the tissue blood flow at the capillary level and plays an important role in transporting gases, such as oxygen and carbon dioxide, and supplying local energy. It can be evaluated by the contrast effect of the organs and tissues by the contrast medium. MRI is performed using a gadolinium contrast medium. Instead of these extrinsic tracers, the ASL method magnetically labels the blood flowing into the organs and uses it as an intrinsic tracer [55]. The “contrast effect” is low; hence, the signal-to-noise ratio is low. Based on this principle, it uses the difference between the magnetically-labeled state and un-magnetically-labeled state; thus, ASL is a subtraction image in principle. With the spread of MR equipment and a static magnetic field of 3.0 Tesla, this imaging method has become clinically feasible. It is a completely non-invasive method because perfusion images can be obtained without using a contrast medium, and it can be said that it is an excellent diagnostic imaging method that takes advantage of MRI.

Although the following studies were not conducted on patients with CKD, their findings suggested an association between nutrition and cerebral blood flow. A study found that prenatal undernutrition was associated with differences in brain perfusion during older age evaluated using ASL [27]. Early nutritional deprivation may cause irreversible damage to the brain and may affect cognitive function in older adults. The effects of flavonoids, dietary nitrate, and caffeine on brain perfusion have also been evaluated using ASL [28,29,30]. ASL also demonstrated clearly that alcohol increased cerebral perfusion [31,32]. It has also been confirmed that the degree of increase in cerebral blood flow varies from region to region [45,46]. In interventional studies, the same subject can be evaluated repeatedly because ASL does not require the use of contrast media.

### 3.8. Magnetic Resonance Elastography

MRE is an imaging method used to evaluate the elasticity and viscosity of living organisms using MRI. The basic principle was reported in the journal Science in 1995 [56], as follows: protons, which are the basis of MR signals, are rotating, and the signals obtained from protons by MR equipment are vector quantities. Usually, the “magnitude” is imaged. This is why they are called “T1-weighted images” or “proton-weighted images.” However, MRE can be said to use the phase information, that is, the “phase contrast-based” MRI technique. In practice, MRE requires ordinary MR equipment, a vibration generator (a speaker that generates air vibrations, a tube that transmits the vibrations, and a pad that attached to the thorax), and software for image analysis. It is mostly used to evaluate the liver. It is used to evaluate fibrosis in liver cirrhosis because vibrations are transmitted faster in hard materials. In nutritional research, MRE is mostly used to evaluate fatty liver and assess the degree of progression of cirrhosis [35].

### 3.9. Magnetic Resonance Spectroscopy

MRS is a non-invasive method for measuring the composition and quantity of compounds in tissues and detecting the amount and spatial distribution of various molecular compounds, which are involved in metabolism [57]. It has already been mentioned that atoms, such as 1H (protons), have a Larmor precession at a specific frequency when placed in a static magnetic field. This frequency depends on the magnitude of the static magnetic field. Even for the same proton, the magnitude of the static magnetic field acting on the proton will differ owing to differences in electron distribution caused by the influence of surrounding atoms and substituents (this is called the “shielding effect”). As a result, protons in water and those in lactic acid, for example, have different resonance frequencies, even if the static magnetic field added by the apparatus is the same. This difference in frequency is called a chemical shift. When MR signals are collected for protons, the signals from protons in water molecules overwhelmingly dominate the entire signal. However, by precisely resolving the frequency, signals from protons in other compounds can be captured. This is the basic principle of MRS. Unlike NMR systems that analyze compounds, medical MR equipment has a small static magnetic field; thus, 1H-MRS is mainly used for protons. In proton MRS (1H-MRS), the targets of measurement are creatine, choline, N-acetyl-acetate, citrate, lactate, and lipids [58]. In addition, when using a high-magnetic field device, it is expected that it will be possible to measure substances related to energy metabolism, such as ATP, based on signals from 31P (phosphorus).

Several studies, not limited to patients with chronic kidney disease, utilizing the MRS, have reported about the relationship between brain metabolism and nutrition. Normal brain cells depend on glucose metabolism, yet they have the flexibility to switch to the usage of ketone bodies during caloric restriction. In contrast, tumor cells lack genomic and metabolic flexibility and are largely dependent on glucose levels. Hence, a ketogenic diet (KD) has been suggested as a therapeutic option for malignant brain cancer. A 1H-MRS was, in fact, able to visualize the effects of treatment in patients with brain tumors who adhered to a KD [36]. Moreover, a 1H-MRS detected metabolic effects in different brain regions caused by food consumption [37]. It was also reported that dairy food consumption was associated with cerebral glutathione concentrations in older adults [38]. These data showed that the 1H-MRS is a non-invasive tool suitable for nutritional assessment. Cheng et al. used a 1H-MRS and a dual-echo in-phase and out-phase MRI (dual-echo MRI) to assess the effects of dietary nutrient intake on hepatic lipid content [39]. In their conclusion, hepatic fat content was associated with high-energy, high-fat, and high-saturated fatty acid intake, quantified by 1H-MRS and dual-echo MRI. The method of simultaneously evaluating the same organ under multiple conditions is also called “multiparametric MRI” and is currently attracting attention as a multifaceted evaluation method unique to MRI that is not possible with X-ray CT.

### 3.10. Blood Oxygenation Level-Dependent–MRI

BOLD MRI is an MRI technique originally used to study the active areas of the brain [59]. Many studies have been reported on areas of brain activity related to taste, smell, and food perception, and many of the papers are closer to neuroscience research than nutrition research. As for reports related to nutritional science, there are many studies examining the relationship between dietary content, overeating, obesity, and central nervous system, especially brain function.

In a study, functional BOLD-MRI maps of the motor area were obtained from a patient receiving hemodialysis before and after a hemodialysis session. This report demonstrated a decrease in the maximum intensity of BOLD response, while the BOLD area increased in the primary motor cortex after hemodialysis. These changes were involved with oxidative stress levels. It is known that oxidative stress is systematically increased in patients on hemodialysis after the hemodialysis process. The BOLD-fMRI shows a remarkable sensitivity to brain plasticity and reorganization of the functional control of the studied cortical area. The results also confirmed the superiority of the BOLD-MRI compared with the biological method used for assessing oxidative stress generated by hemodialysis [40].

A report, not about patients receiving dialysis, was based on the fact that the human brain is essential for regulating the intake of food and beverages by balancing energy homeostasis with reward perception. Using BOLD MRI, the effects of ingestion of glucose, fructose, sucrose, and sucralose (a non-caloric artificial sweetener) on the magnitude of brain responses was investigated. The results demonstrated that, while the brain responded directly and readily to glucose as a preferred source of energy in the brain, it may not have responded as efficiently to other sugars [41]. Other research results on the relationship between eating behavior and brain activity using BOLD MRI have also been reported [42,43] and are considered to be important basic research results for effective diet planning from the aspects of brain science and behavior.

## 4. Conclusions

Relevant observations from multiple published reports on the plethora of uses of MRI in nutritional science research have been summarized in this article, along with an overview of the principles of each imaging method. For morphological assessment, MRI has been widely reported to be useful for body composition analysis and can provide comprehensive and quantitative assessment values. Since it is a completely non-invasive method that does not use ionizing radiation, it is expected to have an increasingly wider application than X-ray CT. DTI was frequently used in conjunction with conventional MRI to evaluate the structure of cerebral white matter. ASL was reported to be an important evaluation method for cerebral blood flow because it does not use contrast media and has a relatively wide evaluation range. Although the constituents that can be evaluated with MRS are limited, the evaluation of metabolic products has been demonstrated to provide important information that cannot be obtained using urine or blood tests, because MRS involves location information. BOLD MRI, referred to as an fMRI of the brain, has been found to provide information on the neuroscience of eating behavior and cognition related to eating, olfaction, vision, and appetite. Nutritional science is a multidisciplinary and comprehensive field of research; therefore, there are many different applications of MRI. The use of MRI as a candidate evaluation tool is recommended for future research.

## Figures and Tables

**Table 1:** A summary of the various MRI methods used in nutrition-related clinical studies reviewed in this article.

Imaging Procedure	Primary Evaluation Objective	Example of Use	Nutrition-Related Clinical Study Included
Conventional MRI	Structural Evaluation based on proton distribution.	Measurement of the location and volume of adipose tissue and muscle tissue. Assessment of the volume of the brain and other parenchymal organs.	Addeman, B.T. et al. [2] Molfino, A. et al. [3] Fischer, K. et al. [4] Maskarinec, G. et al. [5] Ishihara, S. et al. [6] Abe, T. et al. [7] Spinnato, P. et al. [8] Carrero, J.J. et al. [9] Johansen, K.L. et al. [10] Morrell, G.R. et al. [11] Gamboa, J.L. et al. [12] Martinson, M. et al. [13] Delgado, C. et al. [14] Wells, C.I. et al. [15] Salinari, S. et al. [16] Carter, M. et al. [17] Yang, Y.X. et al. [18] de van der Schueren, M.A. et al. [19] Bourdel-Marchasson, I. et al. [20]
Diffusion Tensor Imaging	Evaluation of microstructure in the tissue based on the anisotropy of thermal diffusion of protons.	Assessment of the degree of degeneration and development of cerebral white matter based on nerve fiber structure.	Drew, D.A. et al. [21] Witte, A.V. et al. [22] Ottolini, K.M. et al. [23] Blesa, M. et al. [24] Coviello, C. et al. [25] Shen, Q. et al. [26]
Arterial Spin Labeling	Evaluation of tissue perfusion using magnetically labeled protons as an endogenous tracer	Assessment of the changes in regional blood flow in the brain	de Rooij, S.R. et al. [27] Lamport, D.J. et al. [28] Presley, T.D. et al. [29] Vidyasagar, R. et al. [30] Rickenbacher, E. et al. [31] Khalili-Mahani, N. et al. [32] Strang, N.M. et al. [33] Marxen, M. et al. [34]
Magnetic Resonance Elastography	Evaluation of organ elasticity based on strain when the organ is vibrated.	Evaluation of progression of liver diseases, such as cirrhosis, fatty liver, etc. It mainly evaluates changes due to fibrosis of organs.	Furlan, A. et al. [35]
Magnetic Resonance Spectroscopy	Evaluation of the amount and spatial distribution of various molecular compounds, based on the principles of NMR.	Evaluation of various metabolites in the brain, including N-acetyl aspartate, γ-aminobutyric acid, glutamine, and lactate, by 1H-MRS.	Artzi, M. et al. [36] Park, Y. et al. [37] Choi, I.Y. et al. [38] Cheng, Y. et al. [39]
Blood Oxygenation Level Dependent-MRI	Assessment of brain activation sites via increased regional cerebral blood flow from changes in deoxyhemoglobin concentration.	Identification of changes in activity and functional areas of the brain associated with appetite, nutritional intake, and eating behavior.	Belaich, R. et al. [40] van Opstal, A.M. et al. [41] Hawton, K. et al. [42] Dong, D. et al. [43]

**Table 2:** Association of metabolic syndrome with SEL.

		Prevalence of SEL	*p* Value with Chi-Square Test	Odds Ratio *	95% CI	*p* Value *
Metabolic syndrome	No (*N* = 267)	7.1% (*N* = 19)	<0.01	Ref.		**0.01**
	Yes (*N* = 57)	19.3% (*N* = 11)		3.9	1.5–9.8	

CI, confidence interval; SEL, spinal epidural lipomatosis; Ref., reference value. * Adjusted for age, gender, smoking habit, and drinking history. From Ishihara et al. (2019), with permission.

## Data Availability

Not applicable.
